# Screening of Perinatal Depression Using the Edinburgh Postpartum Depression Scale

**DOI:** 10.1055/s-0042-1743095

**Published:** 2022-03-04

**Authors:** Tenilson Amaral Oliveira, Guilherme Guarany Cardoso Magalhães Luzetti, Márcia Maria Auxiliadora Rosalém, Corintio Mariani Neto

**Affiliations:** 1 Hospital Maternidade Leonor Mendes de Barros, São Paulo, SP, Brazil; 2 Universidade Cidade de São Paulo, Faculdade de Medicina, São Paulo, SP, Brazil

**Keywords:** depression, pregnancy, risk factors, prenatal care, postpartum period, depressão, gravidez, fatores de risco, pré-natal, perído pós-parto

## Abstract

**Objective** To detect depression during pregnancy and in the immediate postpartum period using the Edinburgh postpartum depression scale (EPDS).

**Methods** Cross sectional study of 315 women, aged between 14 and 44 years, who received perinatal care at the Leonor Mendes de Barros Hospital, in São Paulo, between July 1st, 2019 and October 30th, 2020. The cutoff point suggesting depression was ≥ 12.

**Results** The screening indicated 62 (19.7%) patients experiencing depression. Low family income, multiparity, fewer prenatal appointments, antecedents of emotional disorders, dissatisfaction with the pregnancy, poor relationship with the partner, and psychological aggression were all risk factors associated with depression in pregnancy or in the immediate postpartum period. Antecedents of depression and psychology aggression during pregnancy were significant variables for predicting perinatal depression in the multivariate analysis.

**Conclusion** There is a significant association between the occurrence of perinatal depression and the aforementioned psychosocial factors. Screening patients with the EPDS during perinatal and postpartum care could facilitate establishing a line of care to improve the wellbeing of mother and infant.

## Resumo

**Objetivo** Identificar as pacientes com quadro de depressão na gravidez e puerpério imediato através da escala de depressão pós-parto de Edimburgo (EPDS).

**Métodos** Estudo observacional transversal que incluiu 315 mulheres no ciclo grávido-puerperal com idades entre 14 e 44 anos, que foram atendidas no Hospital Maternidade Leonor Mendes de Barros entre 1° de Julho de 2019 e 30 de Outubro de 2020. O ponto de corte utilizado foi ≥ 12 da EPDS para definir se a paciente apresentava depressão.

**Resultados** Encontramos 62 (19,7%) com depressão. Baixa renda familiar, multiparidade, menor número de consultas pré-natal, antecedentes de transtornos emocionais, insatisfação com a gravidez, mau relacionamento com o parceiro, e agressão psicológica foram fatores de risco associados à depressão na gravidez ou no período pós-parto imediato. Antecedentes de depressão e agressão psicológica durante a gravidez foram preditores significativos de depressão perinatal na análise multivariada.

**Conclusão** O estudo mostrou uma associação significativa entre a ocorrência de depressão e os fatores psicossociais acima mencionados. O pré-natal e o puerpério imediato permitem identificar através da EPDS tais pacientes e estabelecer uma linha de cuidados para melhorar o bem-estar materno e do recém-nascido.

## Introduction

The pregnancy-puerperal cycle, either due to psychosocial factors or hormonal changes, is a time of high risk for the development of depression. 1 A study of 1,558 women revealed that 17% of the pregnant women and 18% of women in the immediate puerperium period screened had significant depressive symptoms in late pregnancy. 2 Perinatal depression can lead to a range of consequences, such as the deterioration of maternal care and the distress of the mother-infant relationship. It may also cause adverse outcomes for the child's growth and development if it occurs during the puerperium period. 3 It is vital, therefore, to pay special attention to the diagnosis and early treatment of perinatal depression.

According to Martins, 4 the psychiatric disorders that predominantly affect women in the perinatal period are divided into three categories: deep sadness syndrome, postpartum depression, and postpartum psychosis. The deep sadness syndrome, also known as “baby blues,” begins within the first 2 weeks postpartum, and symptoms may include crying, sadness, increased anxiety, irritability, instability, mood swings, fatigue, and sleep disorders. Postpartum psychosis develops within the first 3 weeks after delivery, a period in which the symptoms are intense and severe, and may include delusions, conferring risk to both mother and child. 5

Perinatal psychiatric disorders usually occur within the first months postpartum, but can happen earlier, 6 with the gradual development of depressive symptoms, which may be mistaken with the deep sadness syndrome after childbirth. 7 Postpartum depression is a psychiatric disorder that causes emotional, behavioral, and physical changes associated with the puerperium. It is estimated that 25 to 35% of women develop depressive symptoms and ∼ 20% may experience depression, with an intensification of symptoms in the 3 ^rd^ trimester of pregnancy. 8

Perinatal depression is multifactorial, involving psychosocial and sociodemographic variables, physiological and biological factors, hereditary predispositions, and hormonal changes. It relates to the body, mind, and lifestyle of the puerperal woman, and is deemed difficult to prevent, as no single strategy has so far been capable of preventing the disorder effectively. 9 Increased stress during pregnancy and delivery has been associated with the etiology of postpartum depression. Consequently, both gestation and postpartum periods require careful analysis to facilitate an early identification of the psychosocial, hormonal, and physiological factors causing depression. 10

As suggested by Salum e Morais et al., 11 the major risk factors for postpartum depression include the lack of support from the partner, family, and friends; low level of education; being a single mother with a high parity; pregnancy at a young age; stress; and low family income. Other factors, such as unwanted pregnancies, primiparous women, preterm births, marital conflicts, and the death of family members or the last infant, also contribute to the adversity. 12

We highlight, thus, the importance of the health professional for the early awareness of the aforementioned factors in the effort to prevent depressive disorders and their consequences in the perinatal period. Difficulties, however, such as lack of time, the stigma related to mental illnesses during pregnancy and in the postpartum, and insufficient or inadequate training in graduate school hinder the early detection by obstetricians. 13 On that account, to reduce the impact of depression in pregnancy and the puerperium on the mother, child, family, and community, it is necessary to discuss the different aspects of the complication in the context of public health.

Developed by Cox et al. 14 in 1987 to assist primary care health professionals to detect postpartum depression disorders, the Edinburgh postpartum depression scale (EPDS) is a screening questionnaire that has been widely used to evaluate symptoms of depression during pregnancy and the puerperium. The test can be completed by the patient in 5 minutes and consists of a scale with 10 items, assessing symptoms related to depression over the preceding 7 days, with scores ranging from 0 to 3 for each item. The final result, therefore, varies from 0 to 30.

The authors evaluated its psychometric properties in the United Kingdom, obtaining a sensitivity of 86% and a specificity of 78%. Different scores (between 9–13 points) were compared in the EPDS, with a good correlation. 14 The scores often used for diagnosis were ≥ 10 or ≥ 12 points. Decreasing the score, the sensitivity increases, but the specificity decreases, causing the occurrence of an elevated false-positive rate. 14 In Brazil, the scale was validated by Santos et al. in 1999. 15 The authors suggest a cutoff point of 11 to 12, with a sensitivity of 72% and a specificity of 88%. The main objective of the present research was to identify patients experiencing depression during pregnancy and the immediate postpartum period using a score ≥ 12 in the EPDS as a cutoff point indicating depression, so as to establish a line of care for the vulnerable group.

## Methods

The cross sectional study included 315 women, aged between 14 and 44 years, who received perinatal care at the Maternity Hospital Leonor Mendes de Barros (HMLMB), in São Paulo, between July 1, 2019 and October 30, 2020. The interview was conducted with 136 pregnant women with more than 28 weeks of gestation, and 176 postpartum women within the first postpartum week. The research protocol was approved by the local research ethics committee before the study began, and all of the women provided written informed consent prior to participating in the interview. After signing the consent form, the patient was enrolled in the study and replied to the interview conducted by one of the authors of study (T. A. O./G. G. C. M. L.). In the interview, the women first answered a questionnaire to identify the major risk factors associated with depression. Afterward, the patient herself filled out the EPDS. The scale was completed in ∼ 5 minutes. The interviewers had been trained in the use of the EPDS, and any doubts that eventually came up were jointly discussed. All such interviews took place in ambulatory or maternity ward settings. The interviewer remained blind to the score or the answers because after EPDS to be completed by the mother then it was placed together with the questionnaire in an envelope.

The cutoff was ≥ 12 to indicate whether the patient had depression. The exclusion criteria were: absence of prenatal care, women pregnant with fetuses with congenital malformations and/or diseases incompatible with life, assisted fertilization, stillbirth, and neo-mortality. The collected data was assessed on Microsoft Office Excel 10 spreadsheets (Microsoft Corp., Redmond, WA, USA), the Epi Info 7 program was used to analyze the frequency distribution of categorical variables, and the Mann-Whitney test was used to analyze the continuous variables. The independent variables were categorized to analyze the association between each independent and outcome variable using a bivariate analysis to calculate the crude odds ratio with 95% confidence interval. Those variables that were associated with a *p* -value < 0.1 in the bivariate analysis were entered into a multivariate logistic regression model to calculate the adjusted Odds Ratio and to eliminate the effects of confounding. Analyses by means of logistic regression were performed using the BioStat version 5.3 software.The statistical significance level was set to *p* < 0.05 for all analyses.

## Results

Among 315 patients, 62 (19.7%) patients scored ≥ 12 in the EPDS screening, experiencing probable perinatal depression. The demographic characteristics of the patients are shown in Table 1 . The age ranged from 14 to 44 years, with the average age of 28.3 ± 6.1 in the group signaling perinatal depression (group 1) and 28.9 ± 6.8 years in the group of women without depression (group 2). There were no significant differences in relation to skin color, marital status, use of contraceptive methods by the couple, occupation of the partner or the pregnant woman, and level of education. There were significant differences related to the average family income, which was lower in the group with depression (group 1).

**Table 1:** Sociodemographic characteristics of the group screened with the Edinburgh postpartum depression scale

Variables	Group 1 ( *n* = 62)	Group 2 ( *n* = 253)	*P* -value
Age average (years)	28.3 ± 6.1	28.9 ± 6.8	0.65
White (%)	26 (41.9)	125 (49.4)	0.29
Married/cohabitation	50 (80.6)	220 (86.9)	0.20
Do not use contraceptives	15 (24.2)	60 (23.7)	0.93
Partner with occupation	48 (77.4)	202 (79.8)	0.67
Mothers with occupation	28 (45.2)	138 (54.5)	0.18
Monthly income average	1,954 ± 1,320	2,393 ± 1,488	0.02
Education ≤ 8 years	10 (16.1)	41 (16.2)	0.98

As seen in Table 2 , depressive symptoms were more frequent in multiparous than in nulliparous women, and the average of prenatal appointments was lower. Group 1 also had a higher incidence of previous depression or emotional disorders. Contrarily, complications associated with the pregnancy and the type of delivery did not present significant differences between the two groups ( Table 2 ).

**Table 2:** Clinical and obstetric variables of the group screened with the Edinburgh postpartum depression scale

Variables	Group 1 ( *n* = 62)	Group 2 ( *n* = 253)	*P* -value
Nulliparous (%)	8 (12.9)	76 (30)	0.01
Average perinatal appointments	7.8 ± 3.2	9.2 ± 3.6	0.01
Pregnancy adversities	26 (43)	78 (30.8)	0.09
Antecedents of depression or psychiatric disorders	26 (41.9)	33 (13)	< 0.01
Vaginal birth	18/27 (66.7)	92/149 (61.7)	0.62

The behavioral aspects of the couple during the third trimester and the first postpartum week were analyzed in Table 3 . Dissatisfaction with the pregnancy was more evident in group 1, in which some patients even acknowledged the wish for abortion in early pregnancy. The lack of a good relationship with the partner was another aspect more frequent in group 1. Contrarily, the support of the partner, both during pregnancy and after childbirth, was not contrasting statistically. Psychological aggression (mistreatment) was also more frequent in group 1, while physical aggression was situated on the threshold of statistical significance. The delivery experience, however, was considered positive for most patients in both groups.

**Table 3:** Behavioral variables of the group screened with the Edinburgh postpartum depression scale

Behavioral variables	Group 1 ( *n* = 62)	Group 2 ( *n* = 253)	*P* -value
Satisfaction with pregnancy	55 (88.7)	244 (96.4)	0.03
Desire to abort in the beginning of the pregnancy	10 (16.1)	13 (5.1)	< 0.01
Bad relationship with the partner	18 (29)	18 (7.1)	< 0.01
Partner's support during pregnancy	52 (83.9)	227 (89.7)	0.19
Partner's support after childbirth	24/27 (88.9)	138/149 (92.6)	0.45
Physical aggression	3 (4.8)	2 (0.8)	0.05
Psychological aggression	14 (22.6)	14 (5.5)	< 0.01
Negative experience during delivery	5/27 (18.5)	13/149 (8.7)	0.16

Standard multiple regressions were performed to differentiate the independent effects of predictor variables on the occurrence of perinatal depression ( Table 4 ). Antecedents of depression or psychiatric disorders and psychology aggression during pregnancy showed to be predictive of perinatal depression development on a multivariable analysis.

**Table 4:** Multivariable analysis showing the risk factors associated with the perinatal depression of the pregnant women ( *n* = 315) screened with the Edinburgh postpartum depression scale

Variables	Coefficient	OR	IC95%	*P* -value
Nuliparous	-0.2312	0.7936	0.33 a 1.93	0.6103
Montly income ≤ R$ 1,800 **	0.5200	1.6820	0.91 a 3.11	0.0972
Satisfaction with pregnancy	0.0482	1.0504	0.25 a 4.43	0.9459
Desire to abort in the beginning of the pregnancy	0.8886	2.4316	0.87 a 6.77	0.0890
Bad relationship with the partner	0.0954	1.1001	0.14 a 8.92	0.9288
Physical aggression	-0.3464	0.7020	0.08 a 6.65	0.7619
Psychological aggression	1.2900	3.6329	1.49 a 8.88	0.0047 *
< 10 appointments **	-0.0204	0.9798	0.53 a 1.82	0.9486
Antecedents of depression or psychiatric disorders	1.1362	3.1149	1.52 a 6.39	0.0019 *

* Statistically significant

** Best cutoff point for analysis

## Discussion

The lower income in group 1 demonstrates the lack of social support for the exercise of motherhood, as concerns, doubts, and domestic conflicts can be triggered due to the lack of economic resources to support the newborn or even the family, also considering that multiparous women are associated with an increased risk of depression. Our study shows that psychosocial risk factors are strongly related with the onset of depression in the sample group, whether due to dissatisfaction with the pregnancy or poor relationship with the partner, even when the experience of childbirth was positive.

The study did not relate physical or hormonal factors as some authors have done, 9 16 searching for a biological cause for depression. It was considered that the antecedent of depression or emotional disorders, more frequent in group 1, makes it unlikely that hormonal changes, typical of pregnancy, will have a preponderant role in depression during the period since all pregnant women had similar hormonal changes, yet only 20% developed depression.

Gauthreaux et al. 17 examined the relationship between the desire to be pregnant and postpartum depression by assessing depressive symptoms. The authors concluded that women who did not wish to become pregnant had a higher risk of developing postpartum depression than women who desired pregnancy. Turkcapar et al. 18 noticed the same correlation, detecting a percentage of women with postpartum depression who were dissatisfied with their pregnancies. Similar to our case study, the authors also concluded that episodes of domestic violence were associated with the group experiencing depression. Among the main risk factors, antecedents of depression and psychological aggression were highlighted. In fact, women who experienced previous history of depression have strong association with depressive symptoms in pregnancy, regardless of ethnicity. 19 In the present study, antecedents of depression or psychiatric disorders along with psychological aggression were found to be independently associated with perinatal depression.

The occurrence of perinatal depression may lead to the discontinuation of breastfeeding, family conflicts, and the neglect of the infant's physical and psychological needs. By compromising the ability to create healthy and stable bonds, the disorder can negatively influence the relationship between mother and child, in addition to cause damage to the psychomotor and language development and, as a result, lead to relevant cognitive and social impairments. 20 Adolescents and children whose mothers had postpartum depression showed an elevated risk of multiple adverse outcomes. 21

This study emphasizes the importance of identifying risk factors based on an individual's subjective experience. The scales for diagnosing depression are tools that facilitate tracking and diagnosing the disorder during prenatal care and the immediate puerperium. The use of validated scales can contribute to the production of new evidence concerning the correlation of risk factors and protective measures during pregnancy. Evidence is necessary to support health professionals in the preventive implementation of an adequate approach to coping with the problem.

Antecedents of depression, physical and emotional stress, either caused by socioeconomic factors such as income or by the lack of support from the partner, are risk factors for perinatal depression. The creation of prenatal programs based on a psychological approach, as stated by some authors, 22 23 24 can contribute to the definition of a preventive line of care focused on perinatal depression. During prenatal care, it is necessary to verify the need for support by assessing the quality of the pregnant women's relationships, contributing to the establishment of positive social relations during the period. Since depression is the most common complication of the perinatal period currently, health professionals, particularly doctors and nurses, play a fundamental role in its early detection as well as intervention. Thus, avoiding the occurrence or worsening of the depressive process and its consequences.

This study suggests that all pregnant women should undergo screening in the third trimester of pregnancy or the postpartum period. The EPDS is a simple and useful instrument for screening, as it presents an easy method for diagnosis. To treat the vulnerable group, alongside screenings, professional references and therapeutic resources are also needed.

## Conclusion

In conclusion, there is a significant association between the occurrence of depression and certain psychosocial factors, notably antecedents of depression and psychological abuse, which were predictive factors in a multivariate analysis. To face this challenge, prenatal care must provide a comprehensive psychological approach to identify and treat the disorder. Offering an appropriate line of care to the vulnerable group will contribute to the improvement of the wellbeing of the mother and the future of the infant.
